# QF-TraderNet: Intraday Trading *via* Deep Reinforcement With Quantum Price Levels Based Profit-And-Loss Control

**DOI:** 10.3389/frai.2021.749878

**Published:** 2021-10-29

**Authors:** Yifu Qiu, Yitao Qiu, Yicong Yuan, Zheng Chen, Raymond Lee

**Affiliations:** Department of Computer Science and Technology, Division of Science and Technology, BNU-HKBU United International College, Zhuhai, China

**Keywords:** quantum finance, quantum price level, reinforcement learning, automatic trading, intelligent trading system

## Abstract

Reinforcement Learning (RL) based machine trading attracts a rich profusion of interest. However, in the existing research, RL in the day-trade task suffers from the noisy financial movement in the short time scale, difficulty in order settlement, and expensive action search in a continuous-value space. This paper introduced an end-to-end RL intraday trading agent, namely QF-TraderNet, based on the quantum finance theory (QFT) and deep reinforcement learning. We proposed a novel design for the intraday RL trader’s action space, inspired by the Quantum Price Levels (QPLs). Our action space design also brings the model a learnable profit-and-loss control strategy. QF-TraderNet composes two neural networks: 1) A long short term memory networks for the feature learning of financial time series; 2) a policy generator network (PGN) for generating the distribution of actions. The profitability and robustness of QF-TraderNet have been verified in multi-type financial datasets, including FOREX, metals, crude oil, and financial indices. The experimental results demonstrate that QF-TraderNet outperforms other baselines in terms of cumulative price returns and Sharpe Ratio, and the robustness in the acceidential market shift.

## 1 Introduction

**FIGURE 1:**
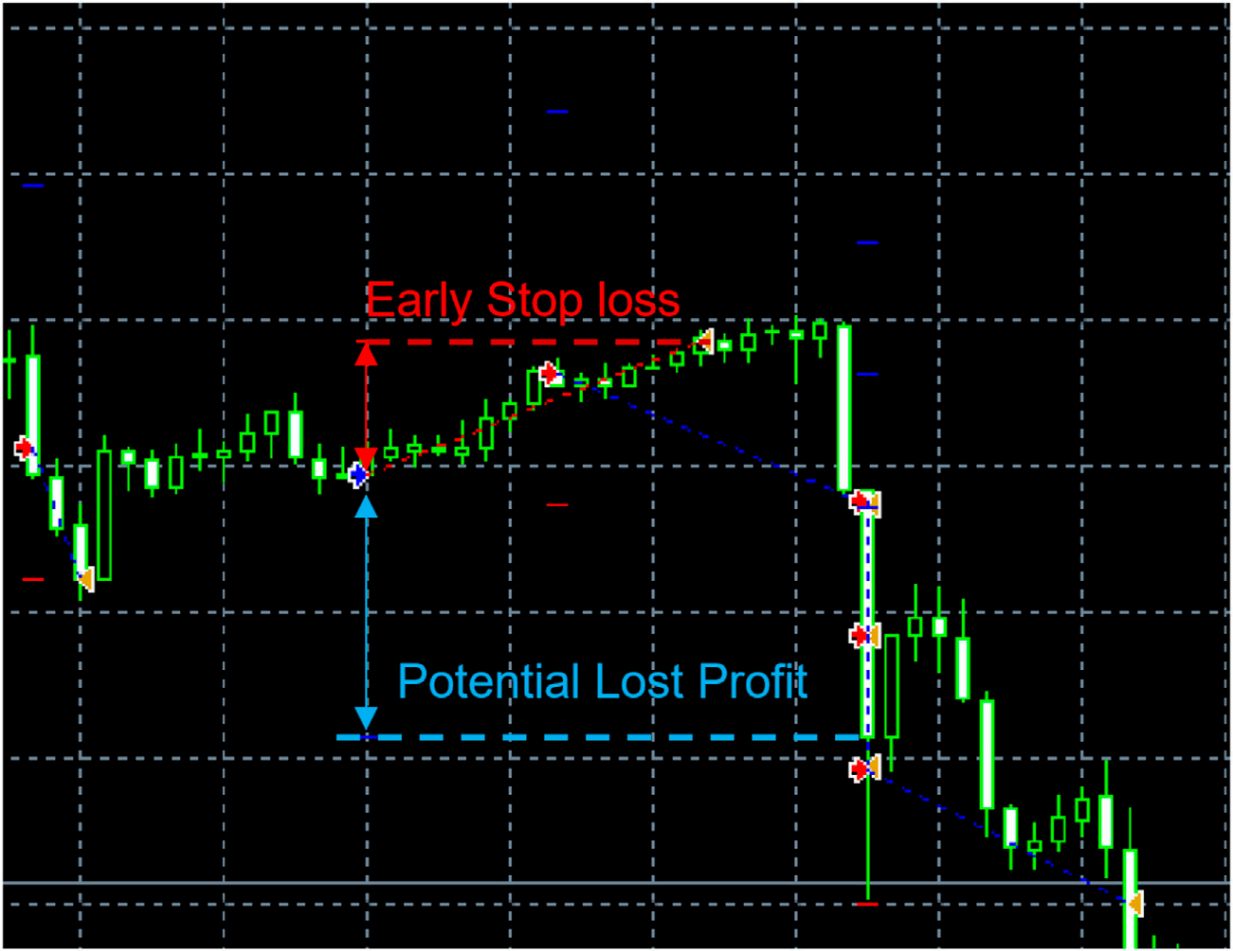
An early-stop loss problem: a short order is early settled (red dash line: SL) before the price drops to the profitable range. Thus, the strategy loses the potential profit (blue double arrow).

Financial trading is an online decision-making process (Deng et al., 2016). Previous works (Moody and Saffell, 1998; Moody and Saffell, 2001; Dempster and Leemans, 2006) demonstrated the Reinforcement Learning (RL) agent’s promising profitability in trading activities. However, traditional RL algorithms face challenges for the intraday trading problem in three aspects: 1) Short-term financial movement is often accompanied by more noisy oscillations. 2) The computational complexity for making decision in daily continuous-value price range. In the *T* + *n* strategy, RL agents are assigned a long, neutral, or short position in each trading day, including the Fuzzy Deep Recurrent Neural Networks (FDRNN) (Deng et al., 2016) and Direct Reinforcement Learning (DRL) (Moody and Saffell, 2001). However, in day trade, i.e., *T* + 0 strategy, the trading task is converted to identify the optimal price to open and close the order. 3) The early stop of orders when applying the intraday strategy. Conventionally, the settlement of orders involved two hyperparameters: Target Profit (TP) and Stop Loss (SL). TP refers to the price to close the activating order and take out the profit if the price moved as expected. SL denotes the price to terminate the transaction and avoid a further loss if the price moved towards a loss direction (e.g., the price dropped down following a long position decision). These two hyperparameters are defined as a fixed shift relative to price to enter the market, as known as, points. If the price touched these two-preset levels, the order will be closed deterministically. An instance of the early-stop order is shown in Figure 1.

**FIGURE 2:**
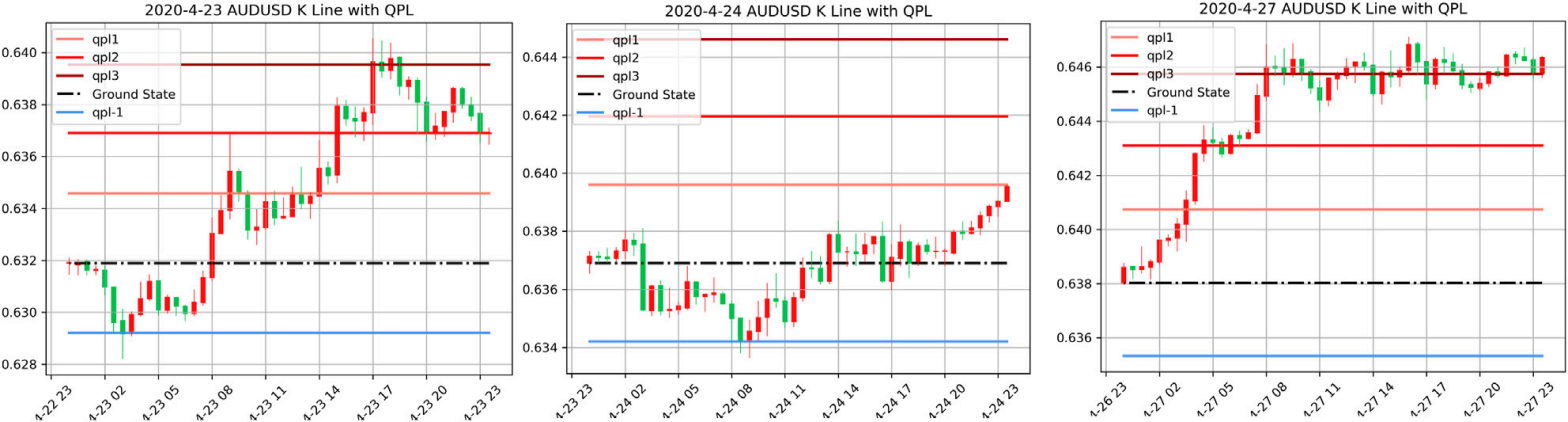
Illustration of AUDUSD’s QPLs in 3 consecutive trading days (23/04/2020–27/04/2020) in 30-min K-line graph. The blue lines represent negative QPLs based on the ground state (black dash line); the red lines are positive QPLs. Line color deepens with the rise of the QPL level *n*.

Focusing on the mentioned challenges, we proposed a deep reinforcement learning-based end-to-end learning model, named QF-TraderNet. Our model directly generates the trading policy to control profit and loss instead of using fixed TP and SL. QF-TraderNet comprises two neural networks with different functions: 1) a Long-short Term Memory (LSTM) networks for extracting the temporal feature in financial time series; 2) a policy generator network (PGN) for generating the distribution of actions (policy) in each state. We especially reference the Quantum Price Levels (QPLs) as illustrated in Figure 2 to design the action space for the RL agent, thus discretizing the price-value space. Our method is inspired by the Quantum Finance Theory that QPLs captures the equilibrium states of price movement on a daily basis (Lee, 2020). We utilize the deep reinforcement learning algorithm to update the trainable parameters of QF-TraderNet iteratively to maximize the cumulative price return.

Experiments on various financial datasets, including the financial indices, metals, crude oil, and FOREX, and comparisons with previous RL and DL-based single-product trading systems have been conducted. Our QF-TraderNet outperforms some state-of-the-art baselines in the profitability evaluated by the cumulative return and the risk-adjusted return (Sharpe ratio), and the robustness facing market turbulence. Our model shows adaptability in the unseen market environment. The generated policy of QF-TraderNet also provides an explainable profit-and-loss order control strategy.

Our main contributions could be summarized as:

• We propose a novel end-to-end daytrade model that directly learns the optimal price level to settle, thus solving the early stop in an implicit stop-loss and target-profit setting.
• We are the first to present RL agent’s action space *via* the daily quantum price level, making the machine day trade tractable.
• Under the same market information perception, we achieve better profitability and robustness than previous state-of-the-art RL based models.

## 2 Related Work

Our work is in line with two sub-tasks: financial feature extraction and transactions based on deep reinforcement learning. We shortly review past studies.

### 2.1 Financial Feature Extraction and Representation

Computational approaches for the applications in financial modeling have attracted much attention in the past. (Peralta and Zareei, 2016). utilized the network model to perform the portfolio planning and selection. Giudici et al. (2021) used volatility spillover decomposition methods to model the relations between two currencies. Resta et al. (2020) conducted a technical analysis-based approach to identify the trading opportunities with specific on cryptocurrency. Among these, the neural networks shows promising ability in learning both the structured and unstructured data. Most of the related works in neural financial modeling were made to the relationship embedding (Li et al., 2019) and forecasting (Wei et al., 2017), option pricing (Pagnottoni, 2019), and forecasting (Neely et al., 2014). The long short-term memory networks (LSTM) (Wei et al., 2017), Elman recurrent neural networks (Wang et al., 2016) were employed in financial time series analysis tasks successfully. Tran et al. (2018) utilized the attention mechanism to refine RNN. (Mohan et al., 2019). leveraged both market and textual information to boost the performance of stock prediction. Some studies also adopted stock embedding to mine the affinity indicators (Chen et al., 2019).

### 2.2 Reinforcement Learning in Trading

Algorithmic trading has been widely studied in its different subareas, including risk control (Pichler et al., 2021), portfolio optimization (Giudici et al., 2020), and trading strategy (Marques and Gomes, 2010; Vella and Ng, 2015; Chen et al., 2021). Nowadays, the AI-based trading, especially, the reinforcement learning-approach, attracts the interest in both academia and industry. Moody and Saffell (2001) proposed a direct reinforcement algorithm to trade and performed a comprehensive comparison between the Q-learning with the policy gradient. Huang et al. (2016) further propose a robust trading agent based on the deep-Q networks (DQN). Deng et al. (2016) utilized the fuzzy logic with a deep learning model to extract the financial feature from noisy time series, which achieved state-of-the-art performance in the single-product trading. Xiong et al. (2018) employed the Deep Deterministic Policy Gradient (DDPG) baesd on the standard actor-critic framework to perform the stock trading. The experiments demonstrated their profitability over the baselines including the min-variance portfolio allocation method and the technical approach based on the Dow Jones Industrial Average (DJIA) index. Wang et al. (2019) employed the RL algorithm to construct the winner and loser portfolio and traded in the buy-winner-sell-loser strategy. However, the intraday trading task for reinforced trading agent are still less addressed, which is mainly because the complexity in designing trading space for frequent trading strategy. We dominantly aim at the efficient intraday trading in our research.

## 3 QF-TraderNet

Daytrade refers to the strategy of taking a position and leaving the market within one trading day. We let our model sends an order when the market is opened every trading day. Based on the observed environment, we train QF-TraderNet to learn the optimal QPL to settle. We will introduce the QPL based action space search and model architecture separately.

### 3.1 Quantum Finance Theory Based Action Space Search

Quantum finance theory elaborated on the relationship between the secondary financial market and the classical-quantum mechanics model (Lee, 2020) (Meng et al., 2015) (Ye and Huang, 2008). QFT proposes an anharmonic oscillator model to embed the interrelationships among financial products. It considers the dynamics of the financial products are affected by the energy field generated by itself and other financial product (Lee, 2020). The energy levels generated from the field of particle regulate the equilibrium states of price movement on a daily basis, which is noted as the daily quantum price level (QPL). QPLs could be viewed as the support or resistance in classical financial analysis indeed. Past studies (Lee, 2019) have shown that QPLs can be used as feature extraction for the financial time series. The procedure of the QPL calculation is given with the following steps.

#### Step 1: Modeling the Potential Energy of Market Movement *via* Four Major Market Participants

Same with the classical quantum mechanics, the *Hamiltonian* in QFT contains the potential term and the volatility term. Founded on the conventional financial analysis, primary market participants include 1) Investor, 2) Speculator, 3) Arbitrageurs, 4) Hedger, and 5) Market maker; however, there is no available chance for Arbitrager to perform effective trading according to the efficient market hypothesis (Lee, 2020). Thus we ignore the arbitrageurs’ effect, and then count the impact of other participants towards the calculation of market potential term:

Market makers provide the facilitator services for other participants, and to absorb the outstanding demand noted as *z*_*σ*_, with absorbability factors *α*_*σ*_. Thus, the excess demand at any instance is given by Δ*z* = *z*_+_ − *z*_−_. The relationship between instantaneous returns $r\left(t\right)=r\left(t,\Delta t\right)=\frac{p\left(t\right)-p\left(t-\Delta t\right)}{p\left(t-\Delta t\right)}$ , and the excess demand could be approximately noted as $r\left(t\right)=\frac{\Delta z}{\gamma }$ , in which *γ* represents the market depth. For an efficient market with the smooth market environment, we assume the absorbability of existing orders with different trading directions will be the same, and the contribution of the market makers is derived as (Lee, 2020),

$$\frac{d\Delta z}{dt}{|}_{MM}=\frac{dz_{+}}{dt}{|}_{MM}-\frac{dz_{-}}{dt}{|}_{MM}$$ (1)

$$=-\alpha_{+}z_{+}+\alpha_{-}z_{-}-\gamma \alpha_{MM}r_{t}$$ (2)

where *σ* denotes the trading position including +: long position, and -: short position. *r*_*t*_ denotes the simultaneous price return respect to time t.

Speculators are trend-following participants with few senses about risk control. Their behavior mainly contributes to the market movement by its dynamic oscillator term. A damping variable *δ* is defined to represent the resistance of trend followers behaviors towards the market. Considering that speculators have less consider risk, there is no high-order anharmonic term regarding the market volatility,

$$\frac{d\Delta z}{dt}{|}_{SP}=-r_{t}\delta {|}_{SP}$$ (3)

Investors have a sense of stopping loss. They are 1) earning profit following the trend, 2) minimizing the risk; thus, we define their potential energy by,

$$\frac{d\Delta z}{dt}{|}_{IV}=r_{t}\left(\delta {|}_{IV}-v{|}_{IV}r_{t}^{2}\right)$$ (4)

where *δ*, *v* stand for the harmonic dynamic term (trend following contribution); and anharmonic term (market volatility), respectively.

Hedger also controls the risk but using sophisticated hedging techniques. Commonly, the reverse trading direction has been performed by Hedgers compared with common Investors, especially for the one-product hedging strategy. Hence, the market dynamic caused by Hedger could be summarized as,

$$\frac{d\Delta z}{dt}{|}_{HG}=-\left(\delta {|}_{HG}-v{|}_{HG}r_{t}^{2}\right)r_{t}$$ (5)

To conclude the equations (3.1) from to (3.4), the simultaneous price return dr/dt could be rewritten as,

$$\frac{dr}{dt}=\gamma \overset{P}{\underset{i=1}{\sum }}\frac{d\Delta z_{i}}{dt}=-\gamma \delta r_{t}+\gamma vr_{t}^{3}$$ (6)

where *P* denotes the number of types of participants inside markets. *δ*, and *v* in Eq. 5 are the summary of each term across all participants models, i.e., *δ* = *γα*_*MM*_ + *δ*_*SP*_ + *δ*_*HG*_ − *δ*_*IV*_, and *v* = *v*_*HG*_ − *v*_*IV*_. Combining *dr*/*dt* with the Brownian price returns described by the Langevin equation, the instantaneous potential energy is modeled with the following equation,

$$V\left(r\right)=\int \left(\gamma \eta \delta r-\gamma \eta vr^{3}\right)dr\approx \frac{\gamma \eta \delta }{2}r^{2}-\frac{\gamma \eta v}{4}r^{4}$$ (7)

where *η* is the damping force factor of the market.

#### Step 2: Modeling the Kinetic Term of Market Movement *via* Price Return

One challenge to model the kinetic term is to replace the displacements in classical particles with an appropriate measurement in finance. Specifically, we replace displacement with price returns *r*(*t*), as *r*(*t*) connects the price change with time unit, which simplifies the Schrödinger equation into the Non-time-dependent one. Hence, the *Hamiltonian* for financial particle could be formulated by,

$$\hat{H}=\frac{\hbar }{2m}\frac{\partial }{\partial r^{2}}+V\left(r\right)$$ (8)

where *ℏ*, *m* denote the plank constant and intrinsic properties of the financial market, such as market capitalization in a stock market. Combining the *Hamiltonian* with the classical Schrödinger equation, the Schrödinger Equation for Quantum Finance Theory (QFSE) comes out with (Lee, 2020),

$$\left[\frac{\hbar }{2m}\frac{d^{2}}{dr^{2}}+\left(\frac{\gamma \eta \delta }{2}r^{2}-\frac{\gamma \eta v}{4}r^{4}\right)\right]\phi \left(r\right)=E\phi \left(r\right)$$ (9)

*E* denotes the particle’s energy levels, which refers to the Quantum Price Levels for the financial particles. The first term $\frac{\hbar }{2m}\frac{d^{2}}{dr^{2}}$ is the kinetic energy term. The second term *V*(*r*) represents the potential energy term, i.e. (3.6), of the quantum finance market. *ϕ*(*r*) is the wave-function of QFSE, which is approximated by the probability density function of historical price return.

#### Step 3: Perform the Action Space Search by Solving the QFSE

According to QFT, if there were no extrinsic incentives such as financial events or the release of critical financial figures, QFPs would remain at their energy levels (i.e., equilibrium states) and perform regular oscillations. If there is an external stimulus, QFPs would absorb or release the quantized energy and jump to other QPLs. Thus, daily QPLs could be viewed as the potential states of the price movements in one trading day. Hence, we employ QPLs as the action candidates in the action space $A=\left\{a_{1},a_{2},\ldots ,a_{A}\right\}$ of QF-TraderNet. The detailed numerical method for solving QFSE and the algorithm for the QPL based action space search is given in the supplementary file.

### 3.2 Deep Feature Learning and Representation by LSTM Networks

LSTM networks show promising performance in the sequential feature learning, as its structural adaptability (Gers et al., 2000). We introduce the LSTM networks to extract the temporal features of the financial series, thus improving the perception in the market status of the policy generation network (PGN).

We use the same *look-back window* in (Wang et al., 2019) with size *W* to split the input sequence ***x*** from the completed series $S=\left(s_{1},s_{2},\ldots ,s_{t},\ldots ,s_{T}\right)$ , i.e., agent evaluates the market status by the time period with size *W*. Hence, the input matrix of LSTM could be noted as $X=\left(x_{1},x_{2},\ldots ,x_{t},\ldots ,x_{T-W+1}\right)$ , where $x_{t}={\left(s_{t-W+w}|w\in \left[1,W\right]\right)}^{T}$ . We design our input vectors ***s***_***t***_ is constituted by: 1) *Opening, highest, lowest and closing prices* for each trading day. Note: the close price in *t* − 1 day might be different with the open price in *t* because of the adjustment of the market outside the trading hours; hence, we consider the entire price variables with four types. 2) *Transaction Volume.* 3) *Moving Average Convergence-Divergence* is a technical indicator to identify the market status. 4) *Relative strength index* is a technical indicator measuring the price momentum. 5) *Bollinger Band (main, upper, and lower)* can be applied to identify the potential price range, consequently observing the market trend (Colby and Meyers, 1988). 6) *KDJ (stochastic oscillator)* is used in short-term oriented trading by the price velocity techniques (Colby and Meyers, 1988).

The principal components analysis (PCA) (Wold et al., 1987) is utilized to compress the series data ***S*** into $\tilde{F}$ dimension and denoise (Wold et al., 1987). Subsequently, the L2 normalization is applied to scale the input features to be in the same magnitude. The preprocessing is calculated as,

$$\tilde{X}=\frac{\underset{F\to \tilde{F}}{PCA}\left(X\right)}{\sqrt{\sum \underset{F\to \tilde{F}}{PCA}{\left(X\right)}^{2}}}$$ (10),

where $\tilde{F}<F$ , and the deep feature learning model could be described as,

$$h_{t}=\underset{\xi }{LSTM}\left(\tilde{x_{t}}\right),t\in \left[0,T-W+1\right]$$ (11)

where ***ξ*** is the trainable parameters for LSTM.

### 3.3 Policy Generator Networks (PGN)

Given the learned feature vector ***h***_***t***_, PGN directly produces the output policy, i.e., the probability of settling order in each + QPL and -QPL, according to the action score $z_{t}^{i}$ produced by a fully-connected networks (FFBPN).

$$z_{t}^{i}=\underset{\theta }{FFBPN}\left(h_{t};W_{\theta },b_{\theta }\right)$$ (12)

where ***θ*** deontes the parameters of FFBPN, with the weighted matrix ***W***_***θ***_ and bias ***b***_***θ***_. Let $a_{t}^{i}$ denotes *i* − *th* action at time *t*. The output policy ***a***_***t***_ is calculated as,

$$a_{t}^{+-}=\frac{exp\left(z_{t}^{i}\right)}{\sum_{a^{i^{\prime }}\in \left[1,A\right]}exp\left(z_{t}^{i^{\prime }}\right)}$$ (13)

in timestep *t*, model takes action *a*_*t*_ by sampling from the policy $a_{t}^{+-}$ comprised of long (+) and short (-) trading direction. $a_{t}^{+-}$ contains *A* dimensions, indicating the number of candidate actions, with the reward of price return $r_{t}^{i}$ for each,

$$r_{t}^{i}=\left\{\begin{array}{ccc} \delta \left(QPL^{\delta i}-p_{t}^{o}\right) & , & \forall QPL^{\delta i}\in \left[p_{t}^{h},p_{t}^{l}\right]\\ \delta \left(p_{t}^{c}-p_{t}^{o}\right) & , & \forall QPL^{\delta i}\notin \left[p_{t}^{h},p_{t}^{l}\right] \end{array}\right.$$ (14)

where *δ* denotes the trading direction: for actions with +QPL as the target price level to settle, the trading will be determined as long buy (*δ* = + 1); for the actions in -QPL, short sell (*δ* = − 1) trading will be performed; and *δ* is 0 when the decision is made to be neutral, as no trading will be made in *t* trading day.

We train our QF-TraderNet with reinforcement learning. The key idea is to maintain a loop with the successive steps: 1) agent *π* aware the environment, 2) *π* make the action, and 3) adjust its behavior to receive more reward until the agent has received its learning goal (Sutton and Barto, 2018). Therefore, for each training episode, a trajectory $\tau =\left\{\left(h_{1},a_{1}\right),\left(h_{2},a_{1}\right),\ldots ,\left(h_{t-1},a_{T}\right)\right\}$ could be defined as the sequence of state-action tuple, with the corresponding return sequence^1^ $r=\left\{r_{1},r_{2},r_{3},\ldots ,r_{T}\right\}$ . The probability of action *Pr* (*action*_*t*_ = *i*) for each QPL is determined by QF-TraderNet as:

$$a_{t}^{i}=Pr\left(action_{t}=QPL^{\left(i\right)}|\tilde{X};\theta ,\xi \right)$$ (15)

$${\left.\begin{array}{c} =\underset{\theta }{\pi_{PGN}}\left(\underset{\xi }{LSTM}\left(\tilde{x_{t}}\right)\right) \end{array}\right|}_{action=i}$$ (16)

let *R*_***τ***_ denotes the cumulative price return for trajectory ***τ***, with $\sum_{t=1}^{T-W+1}r_{t}^{\left(i\right)}=R_{\tau }$ . Then, for all possible explored trajectories, the expectation reward obtained by the RL agent could be evaluated as (Sutton et al., 2000),

$$J_{\pi }\left(\theta ,\xi \right)=\int_{\tau }R_{\tau }\underset{\pi }{Pr\left(\tau ;\theta ,\xi \right)}d\tau$$ (17)

where $\underset{\pi }{Pr\left(\tau |\theta ,\xi \right)}$ is the probability for QF-TraderNet agent *π* with parameters ***θ*** and ***ξ*** to generate trajectory ***τ*** with Monte-Carlo Simulation. Then, the objective is to maximize the expectation of reward, ***θ*** *, ***ξ*** * = *argmax*_***θ,ξ***_ *J* (***θ,ξ***). We substitute objective with its inverse to and use gradient descent to optimize. To avoid the local minimum probelm caused by the multiple postive-reward actions, we use the state-dependent threshold method (Sutton and Barto, 2018) to allow the RL agent perform a more efficient optimization. The detailed gradient calculation is given in the supplementary.

### 3.4 Trading Policy With Learnable Soft Profit and Loss Control

**FIGURE 3:**
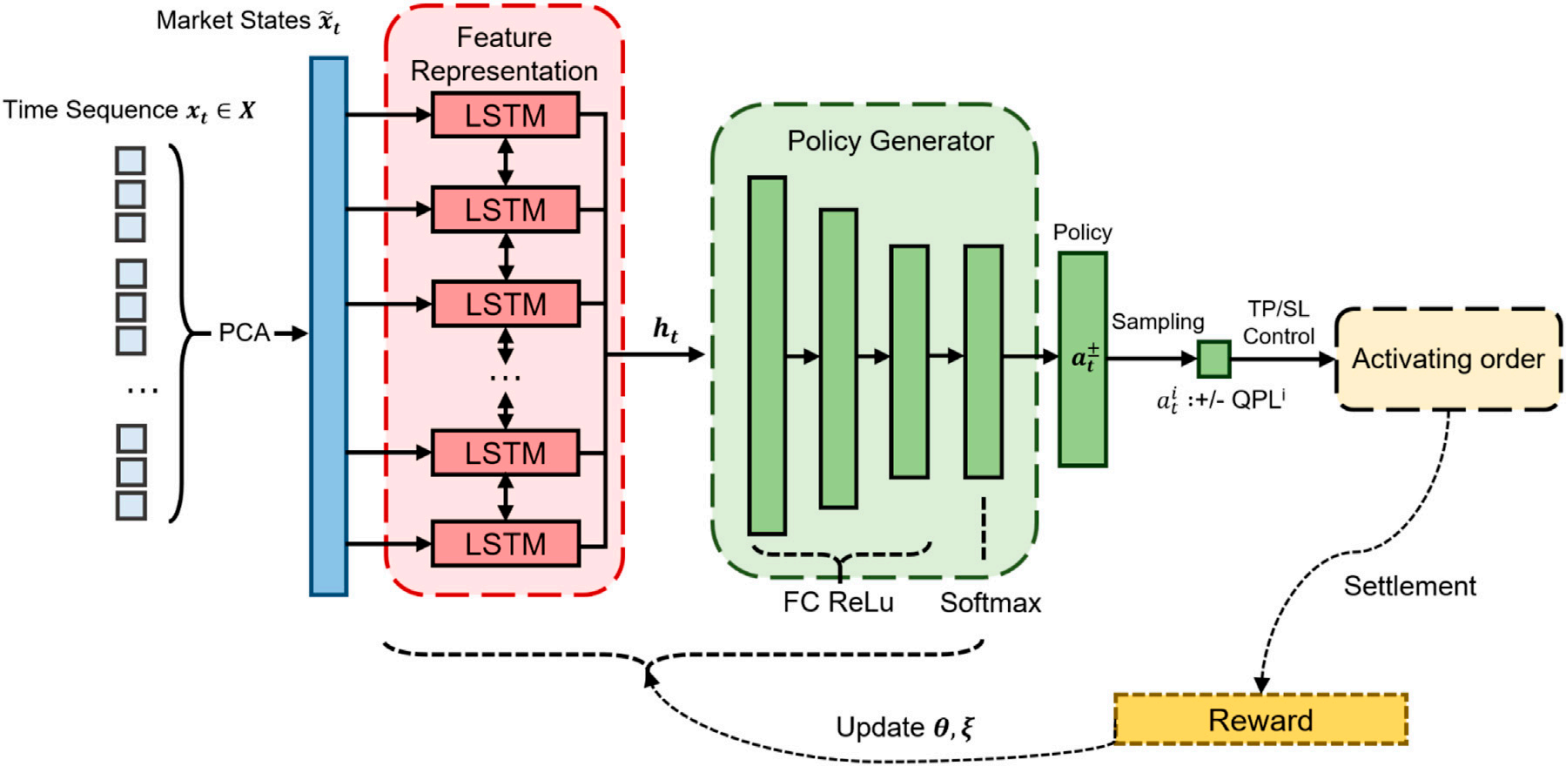
The RL framework for the QF-TraderNet.

In QF-TraderNet, the LSTM networks learn the hidden representation and feed it into PGN; then PGN generates the learned policy to decide the target QPL to settle. As the action is sampled from the generated policy, QF-TraderNet adopts a soft profit-and-loss control strategy rather than the deterministic TP and SL. The overall summary of QF-TraderNet architecture has been shown in Figure 3.

**FIGURE 4:**
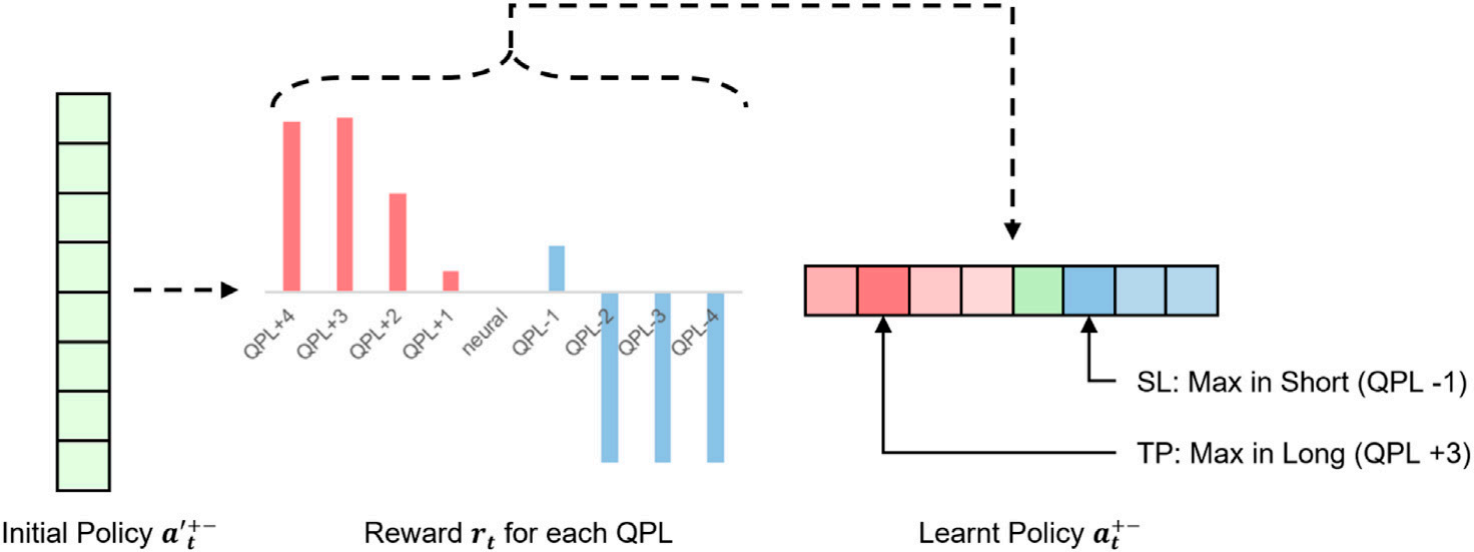
A case study illustrates our profit-and-loss control strategy. The trading policy is uniformly distributed initially. Ideally, our model assigns the +3 QPL action which earns the maximum profit with the largest probability as S-TP. On the short side, −1 QPL can take the most considerable reward, leading to being accredited the maximum probability as S-SL.

An equivalent way to interpret our strategy is that our model trades with long buy if the decision is made in positive QPL. In reverse, short sell transactions will be delivered. Once the trading direction is decided, the target QPL with the maximum probability will be considered as the soft target price (S-TP), and the soft stop loss line will be the QPL with the highest probability in the opposite trading direction. One exemplification is presented in Figure 4.

Since the S-TP and S-SL control is probability-based, when the price touches the stop loss line prematurely, QF-TraderNet will not be forced to do the settlement. It will think whether there is a better target price for settlement in the entire action space. Therefore, the model is more flexible for the SL and TP control in different states, compared with using a couple of preset “hard” hyperparameters.

## 4 Experiments

We conduct the empirical evaluation for our QF-TraderNet in various types of financial datasets. In our experiment, eight datasets from 4 categories are used, including 1) foreign exchange product: Great Britain Pounds vs. United States Dollar (GBPUSD), Australian Dollar vs. United States Dollar (AUDUSD), Euro vs. United States Dollar (EURUSD), United States Dollar vs. Swiss Franc (USDCHF); 2) financial indices: S&P 500 Index (S&P500), Hang Seng Index (HSI); 3) Metal: Silver vs. United States Dollar (XAGUSD), and 4) Crude oil: Oil vs. United States Dollar (OILUSe). The evaluation is conducted from the perspective of earning profits; and the robustness when agents face the unexpected change of market states. We also investigate the impact of different settings of our proposed QPL based action space search for RL trader, and the ablation study of our model.

### 4.1 Experiment Settings

All datasets utilized in experiments are fetched from the free and opened historical data center in *MetaTrader 4*, which is a professional trading platform for the FOREX, financial indices, and other securities. We download the raw time series data, around 2048 trading days, and we split the 90*%* front of data for training and validation. The rest will be utilized as out-of-sample verification, i.e., the continuous series from November 2012 to July 2019, has been spliced to construct the sequential training sample; the rest part is applied as testing and validation. To be noticed, the evaluation period has covered the recent fluctuations in the global financial market caused by the COVID-19 pandemic, which could be utilized as the robustness test when the trading agent is handling the unforeseen market fluctuations. The size of *look-back window* is set at 3, and the metrics regarding price return and Sharpe ratio is daily calculated. In the backtest, initial capital is set to the corresponding currency or asset with a value of 10,000, at a transaction cost with 0.3*%* (Deng et al., 2016). All the experiments are conducted in the single NVIDIA GTX Titan X GPU.

### 4.2 Models Settings

To compare our model with the traditional methods, we select the forecasting based trading model and other state-of-the-art reinforcement learning-based trading agents as the baseline.

• *Market baseline* (Huang et al., 2016). This strategy is used to measure the overall performance of the market during this period *T*, by holding the product consistently.
• *DDR-RNN.* Following the idea of Deep Direct Reinforcement, but we apply the principal component analysis (PCA) to denoise and composes data. We also employ RNN to learn the features, and a two-layer FFBPN as the policy generator rather than the logistic regression in original design. This model can be regarded as the ablation study of QF-TraderNet without the QPL action space search.
• *FCM*, a forecasting model based on RNN trend predictor, consisting of a 7-layer LSTM with 512 hidden dimensions. It trades with a Buy-Winner-Sell-Loser strategy.
• *RF.* Same design with FCM but predict the trend *via* Random Forest.
• *QF-PGN.* QF-PGN is the policy gradient based RL agent with QPL based order control. Single FFBPN is utilized as the policy generator with 3 ReLU layers, and 128 neurons per layer. This model could be admitted as our model without the deep feature representation block.
• *FDRNN* (Deng et al., 2016). A state-of-the-art direct reinforcement RL trader following the one-product trading, by using the fuzzy representation and deep autoencoder to extract the features.

We implement two versions of QF-TraderNet: 1) *QF-TraderNet Lite (QFTN-L)*: 2 layers LSTM with 128-dimensional hidden vector as the feature representation, and 3 layers of policy generator network with 128, 64, 32 neurons per each. The size of action space is 3.2) *QF-TraderNet Ultra (QFTN-U)*: Same architecture with the Lite, but the number of candidate actions is enlarged to 7.

Regarding the training settings, the Adaptive Moment Estimation (ADAM) optimizer with 1,500 training epochs is used for all iterative optimization models at a 0.001 learning rate. For the algorithms requiring PCA, the target dimensions $\tilde{F}$ is set at 4, satisfying the composes matrix has embedded 99.5*%* of the interrelationship of features. In the practical implementation, we directly utilize the four prices as the input for USDCHF, S&P500, XAGUSD, and OILUSe; the normalization step is not performed for the HSI and OILUSe. The reason is that our experimental results show our model can perceive the market state good enough in these settings. For the sake of computational complexity, we remove the extra input features.

### 4.3 Performance in 8 Financial Datasets

**FIGURE 5:**
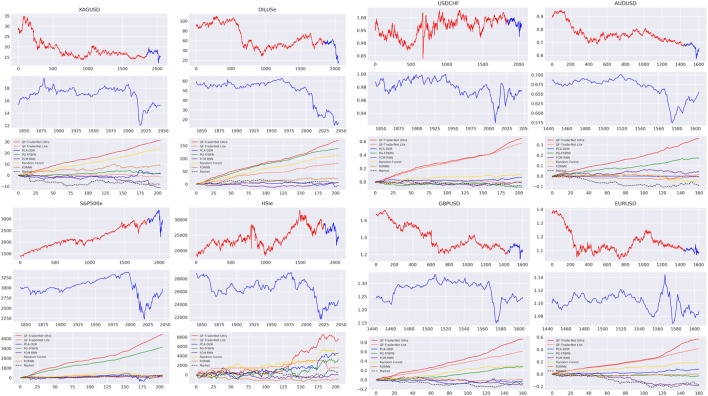
1st panel: Continuous partition for the training and verification data; 2nd panel: Affected by the global economic situation, most datasets showed a downward trend at the testing interval, accompanied by highly irregular oscillations; the 3rd panel: cumulative reward curve for different methods in testing evaluation.

As displayed in Figure 5 and Table 1, we present the evaluation of each trading system’s profitability in 8 datasets, with the metrics of cumulative price return (CPR) and the Sharpe ratio (SR). The CPR is formulated with,

$$CPR=\overset{t}{\underset{1}{\sum }}\left(p_{t}^{\left(holding\right)}-p_{t}^{\left(settlement\right)}\right)$$ (18)

and the Sharpe ratio is calculated by:

$$SR=\frac{Average\left(CPR\right)}{StandardDeviation\left(CPR\right)}$$ (19)

**TABLE 1:** Summary of the main comparison results among all models.

Models	HSI		S&P500		Silver		Crude oil		USDCHF		GBPUSD		EURUSD		AUDUSD	
	CPR	SR	CPR	SR	CPR	SR	CPR	SR	CPR	SR	CPR	SR	CPR	SR	CPR	SR
Market	555.00	0.01	2,122.27	0.05	−12.66	−0.03	−90.79	−0.04	0.19	0.02	−0.07	−0.01	−0.05	−0.01	−0.04	−0.01
RNN-FCM	1,251.78	0.03	361.94	0.09	-11.67	-0.07	6.76	0.02	0.04	0.02	−0.14	−0.07	−0.24	−0.13	0.05	0.04
RF-FCM	3,846.31	0.09	336.27	0.06	23.60	0.91	112.88	1.13	0.11	0.16	0.29	0.33	0.20	0.53	−0.04	−0.07
DDR-RNN	4,505.00	0.10	345.50	0.03	1.53	0.02	−4.57	−0.02	0.07	0.09	-0.02	-0.02	0.08	0.15	<0.01	−0.08
FDRNN	1,536.00	0.04	731.73	0.07	2.80	0.04	−9.38	−0.03	0.08	0.10	0.05	0.04	−0.08	−0.10	0.05	0.12
QF-PGN	3,244.35	0.07	3,133.76	**1.88**	1.94	0.05	138.34	**2.00**	−0.08	−0.11	0.28	0.37	−0.03	−0.05	0.17	0.50
QF-TraderNet Lite	2,779.64	**0.17**	155.66	0.04	1.56	0.04	82.40	0.54	**0.58**	**1.69**	**0.61**	**1.31**	0.20	**0.65**	0.02	0.03
QF-TraderNet Ultra	**8,100.51**	**0.17**	**4,428.00**	1.52	**31.24**	**1.49**	**164.38**	1.44	**0.64**	**1.16**	**0.92**	**1.31**	**0.57**	**1.11**	**0.36**	**0.97**

Bold values indicating the best performance in terms of corresponding metrics.

The result of Market denotes that the market is in a downtrend with high volatility in the evaluating interval, due to the recent global economic fluctuation. The price range in testing is not fully covered in training data in some datasets (crude oil and AUDUSD), which tests the models in an unseen environment. Under these testing conditions, our QFTN-U trained with CPR achieves higher CPR and SR than other comparisons, except the SR in S&P500 and Crude Oil. QFTN-L is also comparable to the baselines. It signifies the profitability and robustness of our QF-TraderNet.

Moreover, QFTN-L, QFTN-U, and the PGN models yield significantly higher CPR and SR than other RL traders without QPL-based actions (DDR-RNN and FDRNN). The ablation study in Table 2 also presents the contribution of each component in detail (Supervised counts from the average of Rf and Fcm), where the QPL actions dramatically contribute to the Sharpe Ratio of our full model. These demonstrates the benefit of trading with QPL to gain considerable profitability and efficient risk-control ability.

**TABLE 2:** Ablation study for QF-TraderNet.

Models	Avg. Sharpe%	Impact
Full Model	1.15	−
QFTN-L: Limit *A* to 3	0.56	−0.59 (−51%)
PGN: - without LSTM	0.59	−0.56 (−49%)
DDR-RNN: - without QPL	0.03	−1.12 (−97%)
Supervised: - without RL	0.19	−0.96 (−83%)

The backtesting results in Table 3 shows the good generalization of the QFTN-U. It is the only strategy for earning a positive profit on almost all datasets, which is because the day-trading strategy are less affected by the market trend, compared with other strategies in long, neutral, and short setting. We also find that the performance of our model in FOREX datasets is significantly better than others. FOREX contains more noise and fluctuations, which indicates the advantages of our models in highly fluctuated products.

**TABLE 3:** Summary for net profit in the backtesting.

	USDCHF	HSI	S&P500	XAGUSD	GBPUSD	EURUSD	AUDUSD	OILUSe
Market	−156.43	−1,505.9	−175	19.29	−28.07	−214.95	−477.53	−7,228.5
FCM	−4,779.2	−5,585.6	−5,656.2	−4,575.5	−3,939.4	−3,685.9	−2,230.1	3,008.8
RF	−5,051.6	**10,589**	−3,302.9	15,536	−284.62	−1,229.5	−1,366.4	66,743
DDR-RNN	−2,727.0	−2,309.5	−3,979.3	**35,248**	−2,298.4	−2,132.2	−3,780.9	−2097.6
FDRNN	−4,543.2	6,204.1	−3,791.8	18,960	−1,619.6	−2,331.1	−3,249.2	4,145.3
QF-PGN	−5,024.0	−4,598.6	3,316.0	−4,203.6	−2,341.2	−3,987.8	−2043.4	**79,433**
QFTN-U	**588.81**	−4,598.6	**10,089**	24,602	**2,499.3**	**399.49**	**538.54**	57,689

Bold values indicating the best performance in terms of corresponding metrics.

### 4.4 QPL-Inspired Intraday Trading Model Analysis

**FIGURE 6:**
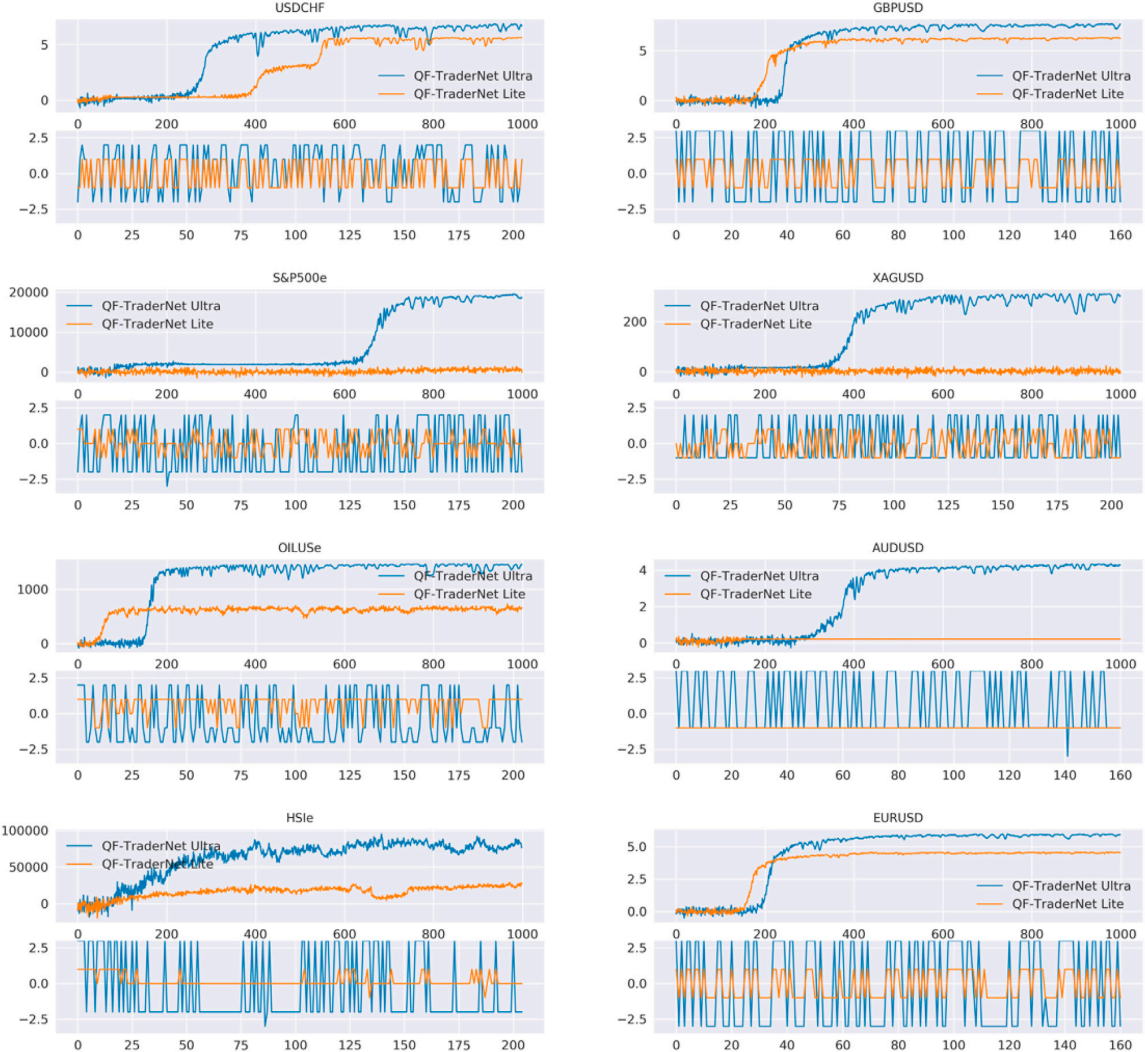
Training curves for different settings in action space size.

We analyze the decision of the QPL-based intraday models in Table 4 as two classifications: 1) predict the optimal QPL to settle; 2) predict the profitable QPL (the QPLs having the same trading direction with the optimal one) to settle. Noticeably, the action space for PGN and QFTN-L is {+1 QPL, Neutral, -1 QPL}, which means that these two classification tasks for them are actually the same. QFTN-7 might have multiple ground truths, as the payoff might be the same while settlement in varied QPLs, thus we only report the accuracy. Table 4 indicates two points: 1) comparing with PGN, our QFTN-L with LSTM as feature extraction has higher accuracy in the optimal QPL selection. The contribution of LSTM to our model can also be proved in the ablation study in Table 2. 2) QFTN-U has less accuracy in optimal QPL prediction compared with QFTN-L, due to the larger action space brings difficulties in decision. Nevertheless, QFTN-U earns higher CPR and SR. We visualize the reward in the training process and the actions made in testing as shown in Figure 6. We analyze that the better performance of QFTN-U is due to the more accurate judgment of trading direction (see their accuracy in the trading direction classification). In addition, QFTN-U can explore its policy in a broader range. When the agent perceives changes in the market environment confidently, it can select the QPL farther than the ground state as the target price for order closing, rather than only the first positive or negative QPL, thereby obtaining more potential payoff, although the action might not be optimal. For instance, if the price is in a substantial increase, agents acquire higher rewards by closing orders at +3 QPL rather than the only positive QPL in QFTN-L’s candidate decisions. According to Figure 6, the trading directions made by two QFTNs are usually the same, but QFTN-U tends to enlarge the levels of selected QPL to obtain more profit. However, the Ultra model needs more training episodes to converge normally (GBPUSD, EURUSD, and OILUSe, etc.). Additionally, the Lite model suffers from the local optimal trap on some datasets (AUDUSD and HSI), in which our model tends to select the same action consistently, e.g., the Lite model keeps delivering a short trade with uniform TP setting in the -1 QPL for AUDUSD.

**TABLE 4:** Decision classification metrices.

	*Optimal QPL Prediction*				*Trading Direction Prediction*			
	Acc.	P	R	F1	Acc.	P	R	F1
Pgn (3x)	0.34	0.25	0.25	0.37	0.34	0.25	0.25	0.37
Qftn-L (3x)	0.56	0.54	0.50	0.50	0.56	0.54	0.50	0.50
Qftn-U (7x)	0.48	—	—	—	**0.80**	**0.78**	**0.78**	**0.82**

Bold values indicating the best performance in terms of corresponding metrics.

### 4.5 Increasing the Size of Action Space

**FIGURE 7:**
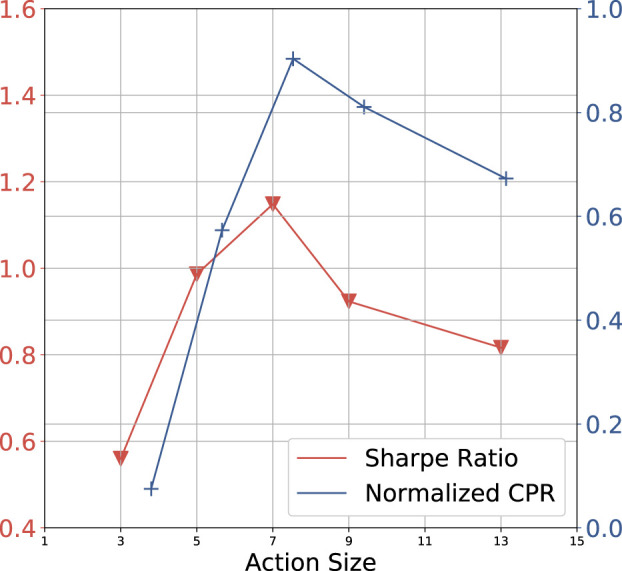
Effects of the different settings in action space size.

In this section, we compare the average CPR and SR among 8 datasets versus different settings of the action space size in Figure 7. We observe that when the size of the action space is less than 7, increasing this parameter has a positive effect on system performance. Especially, Figure 5 shows that our lite model fails in the HSI dataset but the ultra one achieves strong performance. We argue this is because the larger action space can potentially contribute to trading with complex strategies. However, when the number of candidate actions continues to increase, SR and CPR decrease after *A* = 7. We analyze as that the action space of the daytrade model should cover the optimal settlement QPL (global ground truth) within the daily price range ideally. Therefore, if the QPL that brings the maximum reward is not in the model’s action space, enlarging the action space will be more possible to capture the global ground truth. However, if the action space has covered the ground truth already, it is meaningless to continue to expand the action space. On the contrary, a large number of candidate actions can make the decision to be more difficult. We report the results for each dataset in the supplementary.

## 5 Conclusion and Future Work

In this paper, we investigated the Quantum Finance Theory’s application in building an end-to-end day-trade RL trader. With a QPL inspired probabilistic loss-and-profit control for the order settlement, our model substantiate the profitability and robustness in the intraday trading task. Experiments reveal our QF-TraderNet outperforms other baselines. To perform intraday trading, we assumed the ground state in *t*-th day is available for QF-TraderNet in this work. One interesting future work will be combining QF-TraderNet with the state-of-the-art forecasters to perform real-time trading by a predictor-trader framework in which a forecaster predicts the opening price in *t*-th day for our QF-TraderNet to perform trading.

## Data Availability

The original contributions presented in the study are included in the article/Supplementary Material, further inquiries can be directed to the corresponding author.

## Footnotes

1
***r*** in here denotes the reward of RL agent, rather than the previous price return *r*(*t*) in the QPL evaluation
